# Supplementary material for: The Indices of Cardiovascular Magnetic Resonance Derived Atrial Dynamics May Improve the Contemporary Risk Stratification Algorithms in Children with Hypertrophic Cardiomyopathy
Source: J Clin Med. 2021 Feb 8;10(4):650. doi: 10.3390/jcm10040650 (PMC7915130; doi:10.3390/jcm10040650)
Supplement: Supplementary file 1 [file jcm-10-00650-s001.pdf]

Supplementary Materials

Supplementary Table S1. The number of all risk factors in all patients.

**Rick Factor**	
Family history of SCD *n* (%)	12 (21.8)
Massive LV hypertrophy *n* (%)	18 (32.7)
Syncope *n* (%)	16 (29.1)
nsVT *n* (%)	15 (27.2)
Maximal wall thickness z-score *median (IQR)*	7.45 (4.2-14.8)
LA size z-score *median (IQR)*	1.25 (0.17-2.1)

SCD – sudder cardia death, LV – left ventricular, nsVT – non-sustained vetricular tachycardia, LA – left atrial.

Supplementary Table S2. The discriminating performances of HCM Risk Kids and ACC/AHA models with combination with all biatrial volumetric, contractile and displacement markers. HCM RK – HCM Risk Kids model.

**Model**	**AUC**	**95% CI**	**Model**	**AUC**	**95%CI**
HCM RK + LAεa	0.708	0.622-0.846	HCM RK + RAεa	0.629	0.548-0.739
**HCM RK + LAεe**	**0.867**	**0.748-0.949**	HCM RK + RAεe	0.859	0.757-0.949
HCM RK + LAεs	0.633	0.491-0.760	HCM RK + RAεs	0.573	0.461-0.766
HCM RK + LA SRs	0.688	0.547-0.801	HCM RK + RA SRs	0.621	0.502-0.739
HCM RK + LA SRa	0.656	0.515-0.780	HCM RK + RA SRa	0.639	0.572-0.694
HCM RK + LA SRe	0.642	0.505-0.851	HCM RK + RA SRe	0.628	0.554-0.692
HCM RK + LAV max	0.696	0.555-0.754	HCM RK + RAV max	0.651	0.563-0.703
HCM RK + LAV min	0.565	0.423-0.700	HCM RK + RAV min	0.543	0.472-0.611
HCM RK + LVA pac	0.688	0.547-0.807	HCM RK + RVA pac	0.671	0.599-0.739
HCM RK + LAEF total	0.644	0.502-0.770	HCM RK + RAEF total	0.567	0.499-0.640
HCM RK + LAEF passive	0.639	0.496-0.765	HCM RK + RAEF passive	0.594	0.534-0.663
HCM RK + LAEF booster	0.641	0.499-0.767	HCM RK + RAEF booster	0.620	0.555-0.698
ACC/AHA + LAεe	0.753	0.616-0.860	ACC/AHA + RAεe	0.672	0.614-0.730
ACC/AHA + LAεa	0.785	0.652-0.885	ACC/AHA + RAεa	0.664	0.602-0.723
ACC/AHA + LAεs	0.685	0.544-0.804	ACC/AHA + RAεs	0.631	0.586-0.698
ACC/AHA + LA SRs	0.715	0.576-0.829	ACC/AHA + RA SRs	0.689	0.576-0.829
ACC/AHA + LA SRa	0.688	0.547-0.807	ACC/AHA + RA SRa	0.634	0.599-0.674
ACC/AHA + LA SRe	0.734	0.596-0.845	ACC/AHA + RA SRe	0.611	0.587-0.674
ACC/AHA + LAV max	0.698	0.558-0.816	ACC/AHA + RAV max	0.631	0.591-0.699

ACC/AHA + LAV min	0.644	0.502-0.770	ACC/AHA + RAV min	0.672	0.602-0.728
ACC/AHA + LAV pac	0.688	0.547-0.807	ACC/AHA + RVA pac	0.548	0.502-0.611
ACC/AHA + LAEF total	0.679	0.538-0.800	ACC/AHA + RAEF total	0.621	0.572-0.694
ACC/AHA + LAEF passive	0.668	0.527-0.791	ACC/AHA + RAEF passive	0.539	0.449-0.583
ACC/AHA + LAEF booster	0.693	0.553-0.811	ACC/AHA + RAEF booster	0.574	0.501-0.628

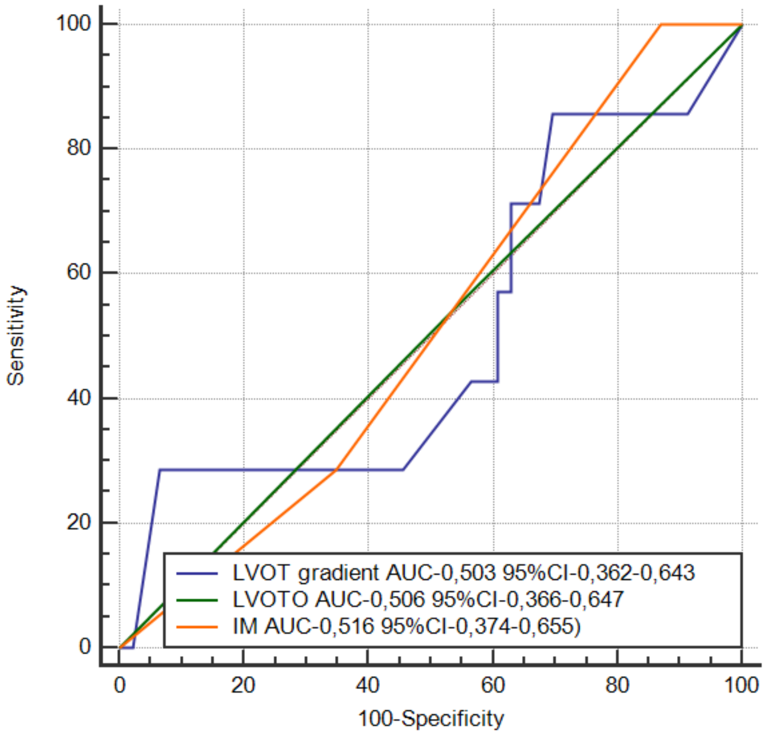

Supplementary Figure S1. Comparison of Receiver Operating Characteristic (ROC) curves for LVOTO (green), LVOT gradient (blue) and mitral regurgitation (orange). LVOTO – left ventricular outflow tract obstruction, LVOT – left ventricular outflow tract, IM- mitral regurgitation.

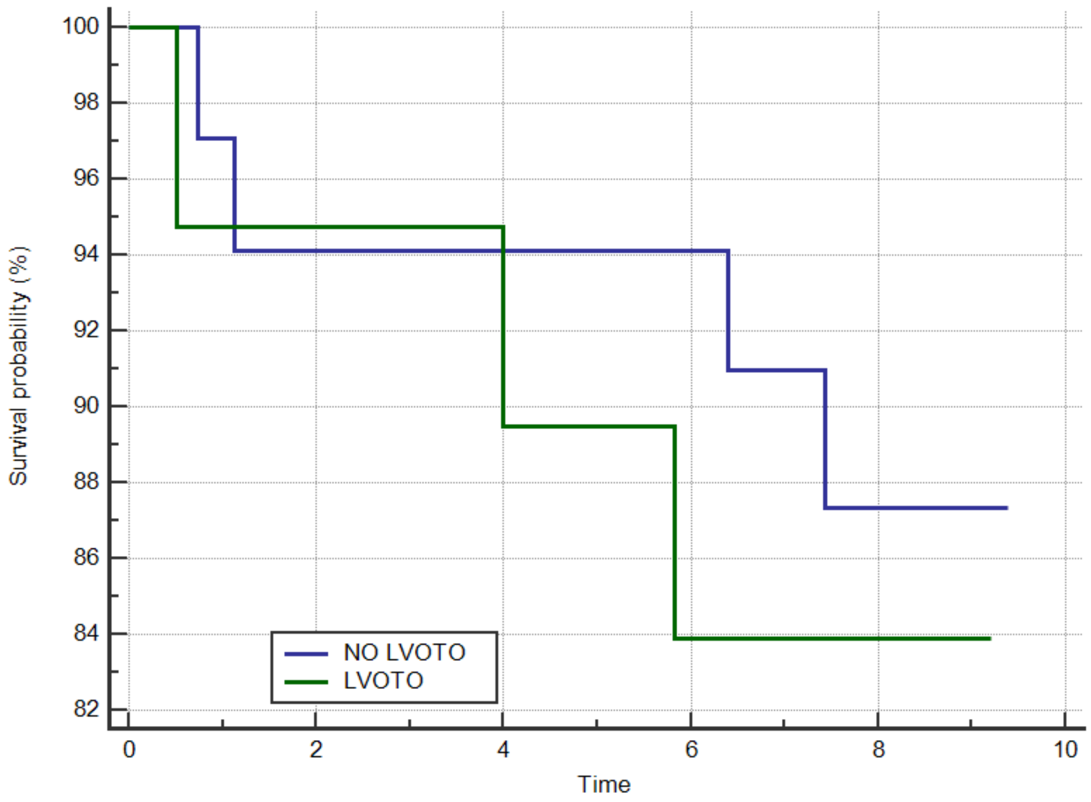

Supplementary Figure S2. Kaplan Maier survival curve comparing survival in children with and without left ventricular outflow tract obstruction. Log rank p = ns. LVOTO - left ventricular outflow tract obstruction.
